# Effect of kinesiophobia on physical activity level in patients with coronary heart disease

**DOI:** 10.3389/fresc.2026.1755286

**Published:** 2026-04-24

**Authors:** Ruo-han Wang, Yan Wang

**Affiliations:** 1 School of Medicine, Kashi University, Kashgar, China; 2 Department of Nursing, School of Medicine, Shihezi University, Shihezi, China

**Keywords:** CHD, IPAQ, kinesiophobia, physical activity, Tampa scale for kinesiophobia heart

## Abstract

**Background:**

Physical activity (PA) is an established effective intervention to improve the prognosis of patients with cardiovascular diseases (CVDs). However, exercise-related barriers frequently hinder PA engagement among this population, limiting its beneficial effects. Given the critical role of PA in cardiac rehabilitation and promising findings from relevant studies, the Tampa Scale for Kinesiophobia (TSK) has been increasingly adopted to assess kinesiophobia in CVD patients. In China, however, understanding of this issue remains inadequate, with related research initiating relatively late.

**Objective:**

This study aimed to determine the levels of kinesiophobia and PA in patients with coronary heart disease (CHD) and evaluate the impact of kinesiophobia on PA levels.

**Design:**

A quantitative, cross-sectional descriptive study was conducted.

**Participants:**

A convenience sample of 401 hospitalized CHD patients was recruited.

**Settings:**

Two Grade A tertiary hospitals in Shihezi City, Xinjiang Uygur Autonomous Region, China.

**Methods:**

Data were collected using a general information questionnaire, the Tampa Scale for Kinesiophobia for Heart (TSK-SV Heart), and the International Physical Activity Questionnaire Short Form (IPAQ-S-C). Kinesiophobia levels, physical activity levels, and their association were analyzed.

**Results:**

The incidence of kinesiophobia among CHD patients was 69.08%. The median PA level was 924 metabolic equivalent minutes per week (MET-min/week). Correlation analysis revealed a strong negative correlation between TSK scores (total and subscale) and PA MET values (r = −0.509, *P* < 0.01). Logistic regression analysis indicated that CHD patients with kinesiophobia were 13.023 times more likely to be in the low PA group compared to those without kinesiophobia.

**Conclusions:**

CHD patients exhibit poor PA levels and an alarmingly high incidence of kinesiophobia. Kinesiophobia scores have a strong negative impact on PA engagement. Healthcare providers should pay close attention to this issue and implement timely interventions to address kinesiophobia in CHD patients.

## 1 Background

Authoritative reports in 2023 indicated that the global prevalence of coronary heart disease (CHD) stood at 239 million. Of all global deaths, 69% were caused by cardiovascular diseases (CVDs) (1). In China, data from the National Center for Cardiovascular Diseases revealed that the prevalence of CHD was 758 per 100,000 population, with a mortality rate of 148.19 per 100,000 population attributed to this disease (2). This situation merits serious attention. The social and economic problems caused by cardiovascular disease are a global problem and a leading cause of death worldwide (3, 4). With the development of modern diagnostics, invasive cardiology and surgical programs, the mortality rate of patients with cardiovascular disease has decreased, and the need for cardiac rehabilitation has also increased (5).Cardiac rehabilitation is generally carried out through a combination of exercise training, secondary cardiovascular disease prevention and psychotherapy (6).

Exercise has emerged as an effective intervention to improve the prognosis of patients with heart disease (7), and its optimal intensity and duration are not only key predictors of cardiovascular disease prevention (8, 9), but also critical factors influencing the therapeutic efficacy in patients with cardiovascular disease (10). As the core component of cardiac rehabilitation, exercise rehabilitation confers multiple physiological and functional benefits to patients with cardiovascular diseases, including improved cardiopulmonary function, enhanced muscle strength, and reduced cardiovascular risk (11–13). Physical activity is a cornerstone of a healthy lifestyle; however, patients with cardiovascular disease often exhibit impaired physical activity levels, which further exacerbates their functional decline and disease burden (14, 15). Psychologically, this impairment is frequently attributed to kinesophobia, a psychological construct characterized by an excessive, irrational fear of engaging in physical activity due to the perceived risk of pain, injury, or disease exacerbation (16, 17).

First conceptualized by Kori et al. (16) in patients with low back pain, kinesiophobia is now increasingly recognized as a harmful barrier to health promotion and disease rehabilitation across various populations, including those with cardiovascular disease. The Tampa Scale for Kinesiophobia (TSK), a well-validated and widely used assessment tool, was developed to quantify and interpret kinesophobia; this scale has been translated into multiple linguistic and cultural versions for global application (18–21), and its utility has been extended to patients with cardiovascular disease (22–24). In China, Lei et al. (25) sinicized the TSK in 2019, confirming its good reliability and validity for use in Chinese populations.

Coronary heart disease (CHD), a major subtype of cardiovascular disease, has attracted widespread clinical and research attention. Previous studies on patients with CHD have consistently shown a high prevalence of kinesophobia (14) and suboptimal physical activity levels (26), which are closely intertwined: kinesophobia often leads to voluntary physical inactivity, while prolonged inactivity further reduces physical fitness and functional capacity, thereby reinforcing kinesophobic tendencies—a vicious cycle that impairs rehabilitation outcomes. Specifically, reduced physical fitness and functional capacity (e.g., decreased exercise tolerance) may heighten patients' perceived risk of adverse events during physical activity, further exacerbating their fear of movement. Conversely, effective management of kinesophobia can promote increased physical activity, which in turn improves physical fitness and functional capacity, breaking this cycle (27).

This study aims to investigate the prevalence of kinesiophobia and the level of physical activity in patients with coronary heart disease, explore the correlation between kinesiophobia (total score and four dimensions) and physical activity level, determine the predictive effect of kinesiophobia on low physical activity in patients with coronary heart disease after adjusting for confounding factors.

Based on existing research, the author proposes the following hypotheses: Kinesiophobia is negatively correlated with physical activity level in patients with coronary heart disease. Compared with those without kinesiophobia, patients with kinesiophobia are more likely to exhibit low physical activity. Furthermore, kinesiophobia serves as an independent influencing factor for low physical activity in this population.

Existing domestic studies have primarily focused on the prevalence and risk factors of kinesiophobia in patients with coronary heart disease, but lack quantitative evidence on the direct impact of kinesiophobia on physical activity levels. Furthermore, few studies have integrated the four-dimensional structure of the TSK-SV Heart Scale to clarify which domain of kinesiophobia is most strongly associated with insufficient physical activity (25). This study fills the above gaps by analyzing a large sample of Chinese patients with coronary heart disease, providing empirical evidence for the mechanism linking kinesiophobia to physical inactivity. The findings will help clinical staff identify high-risk patients with comorbid kinesiophobia and develop targeted interventions to improve physical activity participation and cardiac rehabilitation outcomes.

## 2 Method

### 2.1 Design

This study adopted a cross-sectional descriptive design to explore the correlation between kinesiophobia and physical activity level in patients with coronary heart disease (CHD), and to identify the influencing factors of low physical activity in this population. The combined dataset included 401 participants with coronary heart disease, recruited from two Grade A hospitals in Shihezi City between December 2020 and July 2021 using a convenience sampling method.

### 2.2 Participants

Sample size calculation: The sample size was determined based on the correlation analysis between two continuous variables (kinesiophobia score and physical activity level). The sample size was calculated using the formula for correlation studies: $n\;=\;{\left[\frac{\left(Z_{\alpha /2}+Z_{\beta }\right)}{0.5\times 1n((1+r)/(1-r))}\right]}^{2\;}+\;3$, A two-tailed test was employed with a significance level of *α* = 0.05(Z*_α_*_/2_ = 1.96)and a desired power of 80% (β=0.20,Z_β_=0.84). Based on relevant domestic and international studies, the expected correlation coefficient r between kinesiophobia and physical activity level was set at 0.2. The minimum required sample size was calculated to be 385 participants. To account for a potential 5% rate of invalid questionnaires, a total of 405 questionnaires were distributed, thereby meeting the sample size requirement.

Inclusion criteria: ① Patients with coronary heart disease were diagnosed according to the clinical diagnostic criteria of the European Society of Cardiology in 2016; clinically stable condition, defined as no angina episodes, or no changes in the frequency or nature of angina, within the past month; ② Age ≥18 years old; ③ The patient has no serious physical disease and can walk independently. ④ Patients have no serious mental disorders and cognitive dysfunction, can correctly understand the question and answer; ⑤ The patient gave informed consent and participated voluntarily.

Exclusion criteria: ① The patient was first diagnosed with coronary heart disease, and with unstable angina; ② Patients with acute myocardial infarction; ③ Patients with mental diseases and cognitive disorders; ④ patients who cannot communicate with words; ⑤Patients with lumbar spine or leg problems that are not suitable for exercise; ⑥ Patients who are unwilling to cooperate.

A total of 405 questionnaires were sent out, and 401 were effectively received with effective recovery of 99.01%.All procedures performed were in accordance with the ethical standards of the responsible committee on human experimentation (Institutional Review Board of the First Affiliated Hospital of School of Medicine of Shihezi University).Informed consent was obtained from all patients prior to inclusion in the study.

### 2.3 Methods

#### 2.3.1 Investigation method

The investigators told the CHD patients to fill in the questionnaire according to their real situation, and told the patients that they would protect their personal privacy and relevant information, so as to reduce their concerns and possible bias of the answer. The questionnaire was distributed in a one-to-one manner. After obtaining the informed consent of the respondents, they were asked to answer the questionnaire independently. For patients who could not answer the questionnaire by themselves, investigators assisted them to complete the questionnaire. The investigator explains the items that the patients under investigation cannot understand, but avoids guiding explanations and questions.

#### 2.3.2 Survey tools

##### 2.3.2.1 General information questionnaire

According to the literature reviewed, the General Information questionnaire was designed, including social demographic data, disease-related data and biochemical indicators. Social demographic data include gender, age, education level, occupation, monthly family income, family address, etc. The disease-related data included course of disease, hospitalization times due to coronary heart disease, complications, cardiac function classification（based on New York Heart Association (NYHA) functional classification), complications, stent implantation, etc. anthropometric indicators included height and weight.

##### 2.3.2.2 International physical activity scale short volume Chinese version (IPAQ-S-C)

Hu et al. (28) conducted reliability and validity test on this scale, which confirmed that it had good reliability and validity. There were only 7 items in the short paper. Activity duration was required for each physical activity item, including the number of days the patient participated in activities in the week before hospitalization and the average daily activity duration. Subjects were required to recall their physical activity in the last week and fill in according to their subjective feelings. Physical activity is expressed in the form of metabolic equivalent (MET), and energy expenditure is calculated as follows: Energy expenditure of a certain activity (Met-min-week-1) = metabolic equivalent value of the activity (MET) × average daily activity duration (min) × days of activity/week. MET values were assigned for physical activity based on the metabolic equivalent scale, with 8MET for high physical activity, 4MET for moderate physical activity and 3.3MET for low physical activity (walking).Total energy consumption for physical activity = total light (walking) energy consumption + total energy consumption for medium-intensity physical activity + total energy consumption for high-intensity physical activity. The level of physical activity is divided into three groups: low, medium, and high according to the IPAQ physical activity grading standard. The level of physical activity was divided into three groups (low, medium, and high) Physical activity levels were classified according to the official guidelines of the International Physical Activity Questionnaire Short Form (IPAQ-S-C). The specific criteria were as follows: (1) Low: total physical activity < 600 MET-min/week; (2) Moderate: total physical activity ≥ 600 but < 3,000 MET-min/week; (3) High: total physical activity ≥ 3,000 MET-min/week. To ensure data accuracy, two trained investigators independently calculated the total energy expenditure for each participant. Their results were cross-verified; in cases of significant discrepancy (e.g., difference > 5% or inconsistent values), a third investigator re-calculated the data to confirm the final classification.

##### 2.3.2.3 TSK-SV heart scale

The questionnaire was compiled by Dr. Bäck (23), Lei et al. (25) Sinicized.The scale includes 4 dimensions: risk perception (items 3, 8, 11, 16), fear of exercise(items 1, 7, 9, 13), avoidance of exercise(items 2, 4, 12, 14, 17) and function Disorder (items 5, 6, 10, 15),There are 17 items on the scale, with a total score between 17 and 68 points. Items 4, 8, 12, and 16 are reverse scoring items. The higher the score, the higher the level of kinesophobia. The overall Cronbach's *α* coefficient of the scale is 0.859, and the test-retest reliability is 0.792, which indicates that the scale has good reliability and validity, indicating that this scale is suitable for the assessment of kinesophobia of patients with coronary heart disease in China. The recommended cut-off value for the TSK-SV Heart Scale is 37.A score > 37 indicates the presence of kinesiophobia (high-risk group); A score ≤ 37 indicates no or mild kinesiophobia (low-risk group) (29).

##### 2.3.2.4 Recommended amount for exercise fitness

According to the Essentials of China Cardiac Rehabilitation and Secondary Prevention Guidelines 2018 proposed by the Cardiovascular Professional Committee of the Chinese Society of Rehabilitation Medicine (30), For cardiac rehabilitation patients, a weekly exercise amount of 500∼1000 Met-min can have obvious benefits to their bodies, such as reducing the incidence of coronary heart disease and early mortality. Take 500∼1,000 Met-min-week as the recommended amount of weekly exercise for patients with coronary heart disease.

#### 2.3.3 Statistical analysis

All data were collated and entered into EpiData 3.1 software, and double entry and verification were performed to ensure data accuracy. Statistical analysis was conducted using SPSS 26.0 software. Measurement data were tested for normality first: if they conformed to normal distribution, they were expressed as mean ± standard deviation (x¯ ± s), and t-test or one-way analysis of variance (ANOVA) was used for comparison between groups; if they did not conform to normal distribution, they were expressed as median and interquartile range [M(P25, P75)], and non-parametric test (Mann–Whitney U test or Kruskal–Wallis H test) was used for comparison. Count data were expressed as frequency and percentage (n, %), and chi-square test (*χ*^2^ test) was used for comparison between groups.

The correlation between kinesiophobia (TSK-SV Heart Scale score) and physical activity level (total MET value) was analyzed by Pearson correlation analysis (if normal distribution) or Spearman rank correlation analysis (if non-normal distribution). Multivariate binary logistic regression analysis was used to identify the independent influencing factors of low physical activity in CHD patients, with physical activity level (low vs. medium/high) as the dependent variable, and gender, age, course of disease, kinesiophobia (high-risk vs. low-risk), NYHA classification and other relevant indicators as independent variables. The test level was set at *α* = 0.05 (two-tailed), and *P* < 0.05 was considered statistically significant.

## 3 Results

### 3.1 Participant characteristics

A total of 401 patients with coronary heart disease (CHD) were included in the analysis. The median age was 59 years (range: 28–91 years), and the majority were male (243, 60.60%). Most patients were classified as New York Heart Association (NYHA) functional class I (225, 56.11%) or class II (152, 37.91%). Detailed demographic and clinical characteristics are presented in Table 1.

**Table 1:** Demographic and clinical characteristics of participants (*N* = 401).

Items		N(%)
Gender	Male	243 (60.60)
	Female	158 (39.40)
Age	<45	28 (6.98)
	45∼59	175 (43.64)
	60∼74	124 (30.92)
	≥75	74 (18.45)
Education level	Elementary school and below	112 (27.93)
	junior high school	131 (32.67)
	High school/technical secondary school	77 (19.20)
	College degree and above	81 (20.20)
Family monthly income (yuan)	≤3,000	98 (24.44)
	3,001∼6,000	165 (41.15)
	6,001∼9,000	106 (26.43)
	>9,000	32 (7.98)
Family address	Urban area	220 (54.86)
	Group field	156 (38.90)
	Rural area	19 (4.74)
	Other provinces	6 (1.50)
Living condition	Live alone	53 (13.22)
	Live with spouse	313 (78.05)
	Live with children	35 (8.73)
course of disease(years)	≤0.5	86 (21.45)
	0.5∼1	41 (10.22)
	1∼5	114 (28.43)
	5∼10	67 (16.71)
	≥10	93 (23.19)
Number of hospitalizations due to coronary heart disease (times)	0	114 (28.43)
	1–3	180 (44.89)
	>3	107 (26.68)
Heart function classification	NYHA class I	225 (56.11)
	NYHA class Ⅱ	152 (37.91)
	NYHA class Ⅲ	24 (5.98)
Self-care ability	Patients can take care of themselves completely	386 (96.26)
	Patients can take care of themselves partially	15 (3.74)
Cardiac ejection fraction(EF)	Normal（≥50%）	321 (80.05)
	Abnormal（≤50%）	40 (9.98)
BMI	<18.5	6 (1.50)
	18.5 ≤ BMI < 24.0	91 (22.69)
	24.0 ≤ BMI < 28.0	198 (49.38)
	≥28.0	105 (26.18)
Stent implantation	Yes	142 (35.41)
	No	259 (64.59)
Number of stents	0	259 (64.59)
	1	110 (27.43)
	2	28 (6.98)
	≥3	4 (1.00)
Exercise habits	Have	286 (71.32)
	Not have	115 (28.68)
Complications	Have	287 (71.57)
	Not have	114 (28.43)
Occupation (divided by physical strength)	Light physical labor	317 (79.05)
	Moderate physical labor	15 (3.74)
	Heavy physical labor	69 (17.21)
kinesophobia	Have	124 (30.92)
	Not have	277 (69.08)
Season of investigation	Winter	258(64.34)
	Spring and summer	143(35.66)

*Data for BMI were missing for one participant.

*Exercise habits:defined as engaging in at least 10 min of moderate-intensity physical activity at least three times per week over the past six months.

### 3.2 Prevalence of kinesiophobia and physical inactivity

The median TSK-SV Heart score was 41 (range: 24–61). Based on the established cutoff of >37 points, 277 patients (69.08%) were identified as having kinesiophobia.

Regarding physical activity, the median total energy expenditure was 924 MET-min/week (IQR: 231–1990.5). According to the IPAQ classification, 149 patients (38.16%) had low physical activity levels, 199 (49.62%) had moderate levels, and 53 (13.22%) had high levels. Notably, physical activity was predominantly light-intensity (walking), with a median of 210 (IQR: 60–420) minutes per week. Detailed physical activity data are shown in Table 2.

**Table 2:** Physical activity characteristics of participants (*N* = 401).

Items	Median	IQR (P25, P75)
Total energy expenditure (MET-min/week)	924	(231,1990.5)
High-intensity	0	(0,0)
Moderate-intensity	0	(0,0)
Light-intensity (walking)	693	(198, 1,386)
Total time (min/week)	250	(70, 540)
Time in light-intensity activity	210	(60, 420)

### 3.3 Comparison of physical activity levels by participant characteristics

Univariate analysis revealed significant differences in physical activity levels across several subgroups. Patients who were female, older (≥60 years), living alone, had higher NYHA functional class (II/III), had lower self-care ability, had comorbidities, lacked exercise habits, or had kinesiophobia were significantly more likely to have low physical activity levels (all *P* < 0.05). No significant differences were observed regarding income, disease duration, or BMI (all *P* > 0.05). The complete univariate comparisons are presented in Table 3.

**Table 3:** Univariate analysis of factors associated with physical activity levels in CHD patients (*N* = 401).

Items		Number of cases(%)	Differences in physical activity levels	
			x^2^	P
Gender	Male	243 (60.60)	23.025	0.000
	Female	158 (39.40)		
Age	<45	28 (6.98)	24.394	0.000
	45∼59	175 (43.64)		
	60∼74	124 (30.92)		
	≥75	74 (18.45)		
Education level	Elementary school and below	112 (27.93)	17.417	0.001
	junior high school	131 (32.67)		
	High school/technical secondary school	77 (19.20)		
	College degree and above	81 (20.20)		
Family monthly income (yuan)	≤3,000	98 (24.44)	7.190	0.066
	3,001∼6,000	165 (41.15)		
	6,001∼9,000	106 (26.43)		
	>9,000	32 (7.98)		
Family address	Urban area	220 (54.86)	1.577	0.665
	Group field	156 (38.90)		
	Rural area	19 (4.74)		
	Other provinces	6 (1.50)		
Living condition	Live alone	53 (13.22)	11.490	0.003
	Live with spouse	313 (78.05)		
	Live with children	35 (8.73)		
course of disease(years)	≤0.5	86 (21.45)	6.377	0.173
	0.5∼1	41 (10.22)		
	1∼5	114 (28.43)		
	5∼10	67 (16.71)		
	≥10	93 (23.19)		
Number of hospitalizations due to coronary heart disease (times)	0	114 (28.43)	4.700	0.095
	1–3	180 (44.89)		
	>3	107 (26.68)		
Heart function classification	NYHA class I	225 (56.11)	38.887	0.000
	NYHA class Ⅱ	152 (37.91)		
	NYHA class Ⅲ	24 (5.98)		
Self-care ability	Patients can take care of themselves completely	386 (96.26)	21.060	0.000
	Patients can take care of themselves partially	15 (3.74)		
Cardiac ejection fraction(EF)	Normal（≥50%）	321 (80.05)	2.446	0.118
	Abnormal（≤50%）	40 (9.98)		
BMI	<18.5	6 (1.50)	6.120	0.106
	18.5 ≤ BMI < 24.0	91 (22.69)		
	24.0 ≤ BMI < 28.0	198 (49.38)		
	≥28.0	105 (26.18)		
Stent implantation	Yes	142 (35.41)	2.136	0.144
	No	259 (64.59)		
Number of stents	0	259 (64.59)	3.103	0.376
	1	110 (27.43)		
	2	28 (6.98)		
	≥3	4 (1.00)		
Exercise habits	Have	286 (71.32)	111.781	0.000
	Not have	115 (28.68)		
Complications	Have	287 (71.57)	4.598	0.032
	Not have	114 (28.43)		
Occupation (divided by physical strength)	Light physical labor	317 (79.05)	2.564	0.278
	Moderate physical labor	15 (3.74)		
	Heavy physical labor	69 (17.21)		
kinesophobia	Have	124 (30.92)	84.352	0.000
	Not have	277 (69.08)		
Season of investigation	Winter	258(64.34)	0.796	0.372
	Spring and summer	143(35.66)		

*Data for BMI were missing for one participant.

*Exercise habits:defined as engaging in at least 10 min of moderate-intensity physical activity at least three times per week over the past six months.

### 3.4 Correlation between kinesiophobia and physical activity

Spearman correlation analysis (due to the non-normal distribution of physical activity data) showed that the total TSK score was significantly negatively correlated with physical activity levels (*r* = −0.492, *P* < 0.01). All four dimensions of the TSK scale (risk perception, fear of movement, avoidance of movement, and dysfunction) were also negatively correlated with physical activity, with correlation coefficients ranging from −0.354 to −0.436 (all *P* < 0.01). The correlation matrix is presented in Table 4.

**Table 4:** Correlation matrix between kinesiophobia and physical activity (*N* = 401).

Variable	Total score	Risk perception	Fear of movement	Avoidance of movement	Dysfunction	Physical activity (MET)
Total score for kinesiophobia	1					
Risk perception	0.705**	1				
Fear of movement	0.857**	0.450**	1			
Avoidance of movement	0.874**	0.480**	0.664**	1		
Dysfunction	0.790**	0.492**	0.596**	0.568**	1	
Physical activity (MET)	−0.492**	−0.408**	−0.354**	−0.422**	−0.436**	1

** *p* < 0.01.

### 3.5 Multivariable logistic regression analysis of factors associated with Low physical activity

A binary logistic regression analysis was conducted to identify independent predictors of low physical activity (vs. moderate/high activity). The model included variables that were significant in the univariate analysis (gender, age, education, living status, NYHA class, self-care ability, exercise habits, comorbidities, and kinesiophobia).

The results revealed that kinesiophobia (OR = 13.02, 95% CI: 4.85–34.97, *P* < 0.001) and lack of exercise habits (OR = 11.26, 95% CI: 5.80–21.88, *P* < 0.001) were the strongest independent predictors of being in the low physical activity group. Additionally, higher NYHA functional class (II/III) was significantly associated with low physical activity. The Hosmer-Lemeshow test indicated a good model fit (*P* = 0.095). Detailed results of the regression analysis are shown in Table 5.

**Table 5:** Regression analysis of influencing factors of physical activity level in patients with coronary heart disease.

Factors	β	S.E	Waldχ^2^	*P*	OR（95%CI）
Cardiac function classification			7.788	0.020	
Cardiac function grade I					1
Cardiac function grade II	0.811	0.302	7.193	0.007	2.25 (1.24–4.07)
Cardiac function gradeⅢ	0.811	0.887	5.148	0.042	2.41 (0.66–8.80)
Exercise habits					
Have					1
Not have	2.421	0.339	51.047	<0.001	11.26 (5.80–21.88)
Kinesophobia					
Not have					1
Have	2.567	0.504	25.934	<0.001	13.02 (4.85–34.97)
Age group			7.744	0.052	
<45					1
45∼59	0.743	0.655	1.288	0.256	2.10 (0.58–7.52)
60∼74	1.612	0.711	5.137	0.023	6.01 (1.24–20.19)
≥75	1.548	0.780	3.933	0.047	4.70 (1.02–21.70)

Additionally, higher NYHA functional class (II/III) was significantly associated with low physical activity. Although the overall effect of age did not reach statistical significance (*P* = 0.052), patients aged 60–74 years (OR = 6.01) and ≥75 years (OR = 4.70) showed a trend towards higher odds of low physical activity compared to those <45 years.

## 4 Discussion

### 4.1 Prevalence and dimensional characteristics of kinesiophobia in patients with coronary heart disease

This study found that 69.08% of patients with coronary heart disease (CHD) exhibited kinesiophobia, a prevalence lower than the 76.0% reported in foreign CHD populations (14) but still representing a substantial proportion requiring clinical attention. This discrepancy may be attributed to differences in disease severity, cultural perceptions of illness, or healthcare systems across countries. Chinese patients may underreport psychological distress due to cultural norms that emphasize stoicism, potentially leading to conservative estimates.

The dimensional analysis revealed that “risk perception” (i.e., perception of disease risk associated with heart conditions) scored highest, consistent with findings from Cui et al. (15) and Liu et al. (31). This pattern may be explained by the hypervigilance-avoidance model, wherein patients with CHD develop excessive attentional focus on cardiac symptoms, leading to catastrophic misinterpretation of exercise-induced physiological responses (e.g., increased heart rate) as signs of imminent cardiac events. From a pathophysiological perspective, this hypervigilance activates the sympathetic nervous system, potentially increasing myocardial oxygen demand while simultaneously promoting physical inactivity, creating a vicious cycle that adversely impacts cardiovascular health.

Interestingly, the “avoidance of movement” dimension scored lowest in our sample, diverging from previous studies (15, 31). This discrepancy may reflect regional variations in health literacy or differential exposure to cardiac rehabilitation education. The relatively lower avoidance scores suggest that while patients perceive risks associated with exercise, they do not necessarily translate this perception into active avoidance behaviors, presenting a potential window for intervention.

### 4.2 Physical activity patterns in patients with coronary heart disease

Exercise constitutes the cornerstone of cardiac rehabilitation, yet our findings reveal suboptimal physical activity levels in this CHD population. The median total energy expenditure of 924 MET-min/week was notably lower than values reported in foreign CHD cohorts (32) and domestic studies of post-PCI patients [1386 MET-min/week (33) and 1584 MET-min/week (26)]. This disparity is clinically significant, as insufficient physical activity accelerates the progression of atherosclerosis through multiple mechanisms: impaired endothelial function, reduced nitric oxide bioavailability, and promotion of a pro-inflammatory state characterized by elevated cytokines such as IL-6 and TNF-*α* (34).

Physical activity in our sample was predominantly light-intensity (walking), with a median weekly duration of 210 min. According to IPAQ classification, 62.84% of patients achieved sufficient physical activity levels, a proportion lower than the 73.27% reported by Dąbek et al. (35). When evaluated against the Chinese Cardiac Rehabilitation Guidelines (30), which recommend 500–1,000 MET-min/week for optimal cardiovascular benefits, only 18.20% of patients achieved the recommended range, with 31.41% falling below and 47.38% exceeding this threshold. The high proportion exceeding recommendations warrants particular attention, as excessive exercise intensity without appropriate medical supervision may precipitate adverse cardiac events in vulnerable populations.

This pattern may reflect both cultural preferences for low-impact exercise and underlying fear of more vigorous activities. Walking is often perceived as safe, even among individuals with high kinesiophobia scores, which may explain why the “fear of movement” dimension showed the weakest correlation with physical activity. Nonetheless, the predominance of walking also suggests that patients are not entirely sedentary, offering a behavioral foundation upon which graded exposure interventions can be built.

### 4.3 Association between kinesiophobia and physical activity levels

The regression analysis demonstrated that patients with kinesiophobia were approximately 13 times more likely to be classified into the low physical activity group compared to those without kinesiophobia (OR = 13.02, 95% CI: 4.85–34.97). This odds ratio indicates that, after adjusting for confounders such as age, gender, and NYHA class, the presence of kinesiophobia increases the risk of physical inactivity by more than 13-fold, highlighting its role as a dominant psychological barrier in cardiac rehabilitation.

To contextualize the clinical relevance of this finding, it is useful to compare the magnitude of this effect with other modifiable factors in the same model. Notably, the effect of kinesiophobia (OR = 13.02) was comparable to that of lacking exercise habits (OR = 11.26) and substantially larger than the effects of NYHA class II (OR = 2.25) or III (OR = 2.41). This comparison suggests that the psychological barrier posed by kinesiophobia is at least as important as behavioral factors (exercise habits) and considerably more important than traditional clinical severity indicators (NYHA class) in determining physical activity levels.

From a population perspective, the high prevalence of kinesiophobia (69.08%) combined with its strong effect size translates into a substantial attributable risk. Based on the attributable fraction formula, if a causal relationship is assumed, the proportion of low physical activity attributable to kinesiophobia is approximately 89%. However, considering the realistic efficacy of psychological interventions (typically 50%–60%) and potential barriers to treatment adherence, a more conservative estimate suggests that effectively addressing kinesiophobia could potentially reduce the number of patients with low physical activity by 40%–50%. In absolute terms, this would mean that among the 149 patients currently classified as having low physical activity, approximately 60–75 patients might achieve moderate or high activity levels—a clinically meaningful improvement that warrants investment in targeted interventions.

The magnitude of this association invites comparison with other psychological constructs in cardiovascular research.For instance, a meta-analysis by Barth et al. (36) reported that depression is associated with a 2- to 3-fold increased risk of adverse outcomes in CHD patients, while our findings suggest that kinesiophobia exerts an even stronger effect on a key behavioral outcome—physical activity.This does not diminish the importance of depression but rather underscores that fear-specific constructs may be particularly potent predictors of avoidance behaviors.

The correlation analysis revealed that all four dimensions of kinesiophobia showed significant negative correlations with physical activity levels, with the “dysfunction” dimension exhibiting the strongest association. This finding aligns with the biopsychosocial model of disease, wherein fear-related beliefs about physical activity become self-fulfilling prophecies through multiple pathways (37): psychologically, fear promotes avoidance behaviors (38); physiologically, chronic fear states activate the hypothalamic-pituitary-adrenal axis, increasing cortisol levels and promoting central obesity and insulin resistance (39); socially, activity restriction leads to reduced social participation and diminished quality of life (40).

The particularly strong correlation between the dysfunction dimension and physical inactivity suggests that patients' perceived inability to perform activities—rather than fear of specific movements *per se*—may be the key driver of avoidance. This distinction has important therapeutic implications: interventions targeting perceived competence and self-efficacy (e.g., mastery experiences, vicarious learning) may be more effective than those focused solely on fear reduction.

Notably, the “fear of movement” dimension showed the weakest correlation with physical activity, which may be explained by the predominant mode of exercise in this sample—walking. Among the 286 patients with exercise habits, 85.66% engaged in walking, an activity perceived as low-risk even among individuals who fear more vigorous forms of exercise. However, item-level analysis revealed that endorsement of the statement “My heart problem reminds me when I should stop physical activity so that I don't hurt myself” was particularly high, reflecting a pervasive misconception that symptom-based activity cessation represents optimal self-management rather than a maladaptive avoidance pattern requiring intervention (41).

From a temporal perspective, previous research suggests that kinesiophobia screening is feasible as early as two weeks following acute coronary events, when fear levels demonstrate relative stability (42). However, unlike chronic pain populations where graded exposure to feared activities represents established cognitive-behavioral therapy (43), application of similar principles in CHD populations remains investigational. The persistence of elevated kinesiophobia levels at four-month follow-up (42) suggests that without targeted intervention, fear-related activity avoidance becomes entrenched, perpetuating physical deconditioning and compromising cardiac rehabilitation outcomes.

### 4.4 Clinical implications

The findings of this study have several clinically actionable implications. First, systematic screening for kinesiophobia using the TSK-SV Heart Scale should be integrated into routine cardiac rehabilitation intake assessments. Early identification of at-risk patients would enable targeted interventions before maladaptive avoidance patterns become established. Second, intervention strategies should extend beyond general patient education to incorporate mechanistically grounded approaches:

1. Graded exposure therapy, adapted from chronic pain management protocols, could involve structured progression from symptom-limited activity to gradually increasing exercise intensity under monitored conditions, with cognitive restructuring targeting catastrophic misinterpretations of exercise-induced physiological responses.
2. Heart rate variability biofeedback may help patients recognize that exercise-related tachycardia represents appropriate physiological adaptation rather than pathological decompensation.
3. Cognitive restructuring should specifically address the misconception that symptom-based activity cessation is protective, reframing gradual activity progression as essential for cardiovascular health. Third, the observation that 47.38% of patients exceeded recommended activity levels suggests that some patients may benefit from guidance regarding appropriate exercise titration rather than solely encouragement to increase activity.

### 4.5 Limitations and strengths

Several limitations of this study should be acknowledged. First, the cross-sectional design precludes causal inferences regarding the relationship between kinesiophobia and physical activity. While our findings demonstrate a robust association, longitudinal studies are needed to establish temporal precedence and examine bidirectional relationships. Second, physical activity was assessed using self-report measures, which may be subject to recall bias and social desirability effects; future studies should incorporate objective measures such as accelerometry. Third, the absence of angina assessment represents a potential confounding variable, as symptom status may influence both fear perceptions and activity levels. Although our inclusion criteria restricted enrollment to clinically stable patients, direct measurement of angina frequency and severity would strengthen future investigations. Fourth, the single-center design may limit generalizability to diverse healthcare settings and geographic regions.

Despite these limitations, this study possesses notable strengths. The sample size (*N* = 401) was adequately powered to detect associations, and the use of validated instruments (TSK-SV Heart Scale, IPAQ) enhances measurement reliability. The comprehensive univariate and multivariable analyses accounted for multiple potential confounders, strengthening confidence in the independent association between kinesiophobia and physical activity. Furthermore, this study provides the first detailed dimensional analysis of kinesiophobia in a Chinese CHD population, offering culturally specific insights for intervention development.

## 5 Conclusions

Kinesiophobia affects approximately 70% of patients with coronary heart disease and is strongly associated with reduced physical activity levels, with affected individuals being 13 times more likely to engage in insufficient activity. The predominance of risk perception as the highest-scoring dimension and the strong correlation between dysfunction scores and physical activity highlight specific targets for intervention. Integration of kinesiophobia screening into cardiac rehabilitation protocols, combined with mechanistically informed interventions such as graded exposure and cognitive restructuring, may enhance physical activity engagement and optimize cardiovascular outcomes in this population. Given the prevalence and impact of kinesiophobia, addressing this psychological barrier should be considered a priority in comprehensive cardiac rehabilitation.

## Data Availability

The original contributions presented in the study are included in the article/supplementary material, further inquiries can be directed to the corresponding author.
