# Correction: Effect of kinesiophobia on physical activity level in patients with coronary heart disease

**DOI:** 10.3389/fresc.2026.1867136

**Published:** 2026-05-11

**Authors:** Ruo-han Wang, Yan Wang

**Affiliations:** 1 School of Medicine, Kashi University, Kashgar, China; 2 Department of Nursing, School of Medicine, Shihezi University, Shihezi, China

**Keywords:** CHD, IPAQ, kinesiophobia, physical activity, Tampa scale for kinesiophobia heart

Affiliation “Department of Nursing, School of Medicine, Shihezi University, Shihezi, China” was omitted for author Ruo-han Wang. This affiliation has now been added for author Ruo-han Wang.

Author Ruo-han Wang was erroneously assigned as corresponding author. The correct corresponding author is Yan Wang.

The original version of this article has been updated.

